# Central Pontine Myelinolysis in Pediatric Diabetic Ketoacidosis

**DOI:** 10.1155/2018/4273971

**Published:** 2018-06-04

**Authors:** Hannah Kinoshita, Leon Grant, Konstantine Xoinis, Prashant J. Purohit

**Affiliations:** ^1^University of Hawaii Pediatric Residency Program, USA; ^2^Department of Pediatric Neurology, Kapiolani Medical Center for Women & Children, USA; ^3^Department of Pediatric Critical Care, Kapiolani Medical Center for Women & Children, USA

## Abstract

Central pontine myelinolysis (CPM) is rarely reported in pediatric patients with diabetic ketoacidosis (DKA). We report this case of a 16-year-old female with new onset diabetes presenting with DKA, who received aggressive fluid resuscitation and sodium bicarbonate in the emergency department. Later she developed altered mental status concerning for cerebral edema and received hyperosmolar therapy with only transient improvement. Soon she became apneic requiring emergent endotracheal intubation. MRI brain showed cerebral edema, CPM, and subdural hemorrhage. She was extubated on day seven and exhibited mild dysmetria, ataxia, unilateral weakness, and neglect. Upon discharge she was able to ambulate with a walker and speak and eat without difficulty. Although less common than cerebral edema, CPM should be considered in DKA patients with acute neurologic deterioration. Fluid and bicarbonate therapy should be individualized, but larger studies would help guide the management. Although poor outcomes are reported in CPM, favorable outcomes are possible.

## 1. Introduction

Diabetic ketoacidosis (DKA) is the presenting feature in 10-70% of cases of new onset diabetes in developed countries [1]. One of the most feared complications of DKA, particularly in children, is the development of cerebral edema (CE). The incidence of CE in DKA is approximately 1%; however, when it occurs, it accounts for 57-87% of DKA related mortality and 10-26% of morbidity [2]. Other neurologic complications of DKA include central nervous system infarction, thrombosis, transient grey and white matter changes, and osmotic demyelination [2–8]. The osmotic demyelination syndrome (ODS) consisting of central and extra pontine myelinolysis is a relatively rare complication of DKA. Central pontine myelinolysis (CPM) was originally described as a complication of rapid correction of hyponatremia, particularly in the setting of malnutrition, alcohol abuse, and liver disease [9, 10]. Demyelination is now believed to occur secondary to rapid osmotic shifts from changes in any osmotically active agent including sodium, potassium, glucose, and ammonia [5–8, 11, 12]. To the best of our knowledge, only five other cases of ODS have been reported in association with pediatric DKA [5, 7, 8, 13, 14].

Since these complications are rare and neurological outcomes can be devastating, maintaining a high index of suspicion is important. Some of the suggested risk factors for the development of CE in DKA are young age, severe acidosis, hypocapnia, elevated blood urea nitrogen (BUN), dehydration, aggressive fluid resuscitation, sodium bicarbonate, and bolus/early insulin administration [15–17]. However, no such definite risk factors for the development of CPM have been identified. Here, we present this case of a 16-year-old female with new onset DKA developing features of both CE and CPM.

## 2. Case Presentation

A previously healthy 16-year-old female visitor from Japan presented to an adult emergency room (ER) with altered mental status and emesis. She was found lying on a bathroom floor in her hotel. There was a history of polyuria and polydipsia for 2 weeks and 8 to 10 kg weight loss during the previous month. Her vital signs upon arrival to the pediatric intensive care unit (PICU) were temperature of 98.1° Fahrenheit, heart rate 110/min, respiratory rate 26/min, blood pressure 140/81 mm of Hg, and 100% oxygen saturations without supplemental oxygen. Her capillary refill time was 4-5 seconds. Her weight was 57 kg. Her Glasgow Coma Scale (GCS) was 13 in the ER, which improved to 15 upon arrival to PICU. Skin rash with infected lesions was noted in her groin. The rest of her physical examination was unremarkable.

Workup in the ER showed hyperglycemia of 472 mg/dL, metabolic acidosis (pH 6.75, pCO2 18.4, pO2 149, HCO3 2.5, base deficit 32.6, anion gap 23.5), ketosis (beta-hydroxybutyrate 11.41), glucosuria, and ketonuria, which were consistent with diabetic ketoacidosis. Her white cell counts were 22.3 k/L, hemoglobin 15.8 g/dL, and hematocrit 47%. The rest of her workup was unremarkable.

The patient received fluid resuscitation with 30 mL/kg of 0.9% normal saline (NS) and 50 mEq of sodium bicarbonate in the ER. Continuous insulin infusion was started at 0.1 unit/kg/hr. After that she was started on intravenous fluids containing 0.45% saline and 75 mEq/L of sodium bicarbonate. This was administered at 150 mL/hr, which was 1.25 times the usual daily maintenance requirement for her weight. A consultation with our PICU was obtained at this stage. No further bicarbonate boluses were given. Her fluids were changed to isotonic fluid with potassium phosphate and potassium chloride and without any bicarbonate. It was administered at the rate of 1.5 times maintenance of daily requirement for weight. She was transferred to the PICU at this stage, where she continued to exhibit severe metabolic acidosis with pH 6.97, pCO2 26.7, HCO3 6.1, and base deficit of 24.5. The DKA management was continued with close monitoring and serial laboratory evaluations.

After few hours of her arrival in the PICU, the patient became disoriented and confused. She was given one time 5 ml/kg of 3% hypertonic saline (HS) due to concern for cerebral edema and she responded well. Four hours later she developed lethargy followed by apnea. She was given additional doses of 3% HS bolus (5 mL/kg) and 0.8 g/kg of 20% mannitol. The hyperosmolar therapy was effective but only transiently; the patient eventually required intubation and mechanical ventilation for recurrent apnea.

CT head obtained at that time showed a thin, right parietal subdural hemorrhage without any evidence of edema or mass effect. Of note, CT scan was obtained after the initiation of hyperosmolar therapy.

Hyperosmolar therapy was continued afterwards with 3% HS and 20% mannitol. It was guided with close monitoring of renal function including serum sodium and serum osmolarity. The doses and other parameters were maintained per standard of care [18, 19].

The patient's serum sodium was 135 upon arrival in the ER and 143 at around 11 hrs before the initiation of hyperosmolar therapy and reached to 160 at 71 hrs when hyperosmolar therapy was discontinued. It was normalized to 143 at 131 hrs of admission. Her osmolarity was 298, 305, 335, and 299 at those timings (sodium in mmol/L and osmolarity in msom/kg). The rate of glucose reduction was < 50 mg/dl/hr.

**Figure 1:**
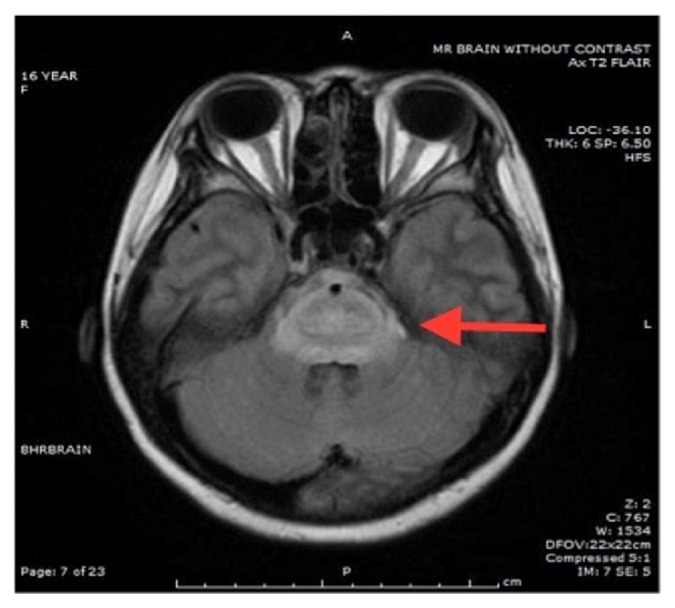
T2-FLAIR Axial image showing diffuse pontine edema.

**Figure 2:**
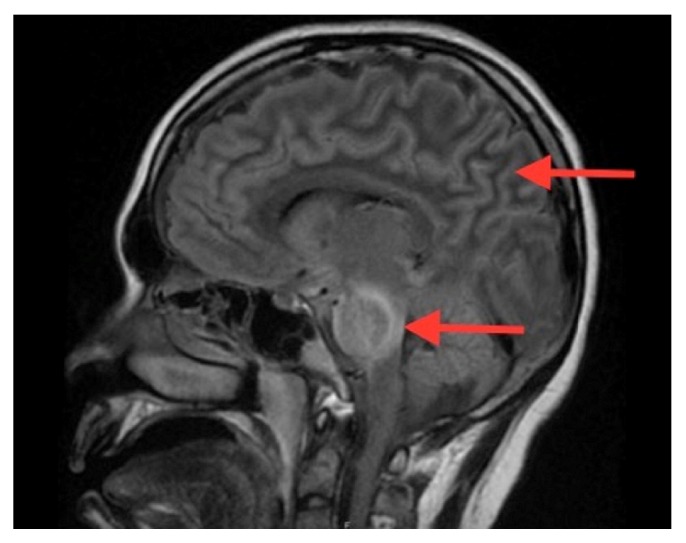
T2-FLAIR Sagittal image showing diffuse pontine edema and cerebral edema.

**Figure 3:**
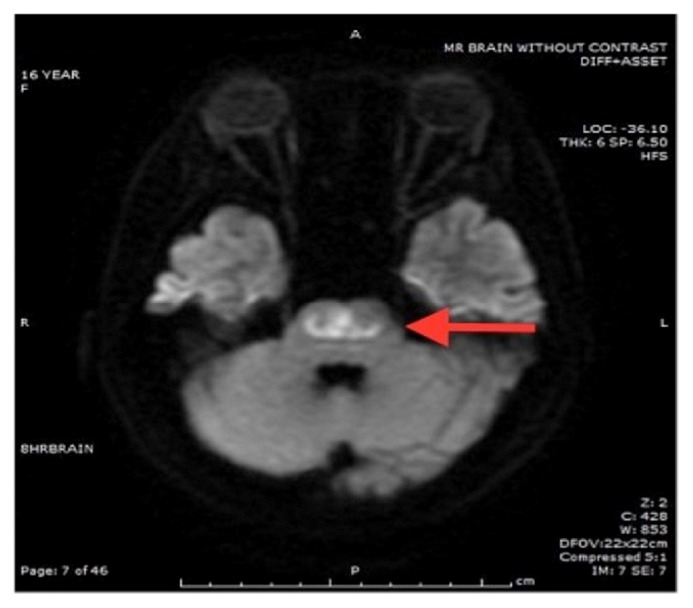
Restricted diffusion in pons with relative sparing of corticospinal tract.

**Figure 4:**
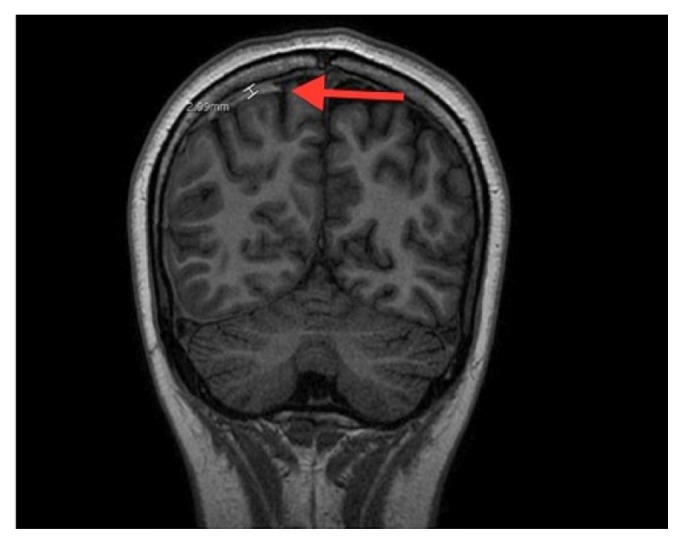
FSPGR sequence, coronal image of brain showing right parietal subdural hemorrhage.

MRI brain obtained at 60 hrs of admission showed mild edema of the cortex and sulci and diffuse edema of the pons and midbrain with restricted diffusion in the pons consistent with central pontine myelinolysis also known as osmotic demyelination syndrome. There was no significant change in the subdural hemorrhage (Figures 1–4). MR angiogram showed no evidence of vessel abnormality.

The patient required continued mechanical ventilation and further management in the PICU. She became more responsive on day 5, and her support was gradually weaned until extubation on day 7. At that time she exhibited only mild residual dysmetria, trivial ataxia, mild left sided weakness, and neglect. All deficits subsequently improved.

Her course was complicated by an isolated focal seizure that responded to standard antiepileptic medication. She also developed bilateral upper extremity deep venous thromboses (DVT). Hematology workup showed no underlying hypercoagulability. Thus the DVT was considered as another complication of DKA [20–22].

Her overall hospital length of stay was 14 days, and by the date of discharge she was able to ambulate with a walker and speak and eat without difficulty.

## 3. Discussion

This case report portrays two potentially fatal complications of DKA, which are CE and CPM. It is possible that our patient developed these neurologic sequalae due to combination of prolonged cerebral edema and central pontine myelinolysis. It is unclear if our patient developed these complications due to severity of illness, therapies administered, or a combination of these elements.

The major indicators of severity of illness were acidosis (pH of 6.75 on presentation) and the history of 8-10 kg weight loss in one month. Thereafter she was exposed to multiple risk factors including rapid initial rehydration, bicarbonate infusion, and hyperosmolar therapy.

Literature on bicarbonate therapy in the management of diabetic ketoacidosis is diverse. One study supports bicarbonate administration in the adult population for blood pH < 7.20 or in cases of fluid refractory hemodynamic instability [23]. Another adult study found no usefulness of sodium bicarbonate therapy in this population [24]. Pediatric literature is suggestive of increased risk of complications including CE with bicarbonate therapy [2, 3, 15, 17, 25]. The ISPAD [3] recommends use of sodium bicarbonate in case of life threatening hyperkalemia and ESCS [2] in case of profound acidosis affecting action of epinephrine during resuscitation. It is possible that bicarbonate administration contributed to the development of cerebral edema in our case.

There are studies suggesting the association of cerebral edema with aggressive fluid resuscitation in the pediatric DKA patients [16, 26]. Literature also exists which failed to demonstrate this association [27, 28]. The European Society Consensus Statement (ESCS) on DKA and International Society for Pediatric and Adolescent Diabetes (ISPAD) recommend a fluid bolus of 10 to 20 mL/kg over 1-2 hours with repeating if necessary [2, 3]. Our patient received a 30 mL/kg fluid bolus, and it might have contributed to the development of CE. However, further larger studies would be helpful to establish this association.

The usual practice at our institution is to follow the recommendations from ESCS on DKA regarding fluid and bicarbonate administration [2]. However, this patient was initially managed in the ER of another institution.

Hyperosmolar therapy could have contributed to the development of CPM in our case. It is also noteworthy that 3% saline was our first choice of hyperosmolar therapy, which is different from the recommendations from ISPAD and ESCS on DKA. Both these recommendations are based on a retrospective cohort study [29], which actually concluded equipoise on the choice of hyperosmolar agent because of certain limitation of their data. In that study, the mortality was higher in cases treated with HS as a single agent versus mannitol as a single agent. Our patient received combined therapy of HS and mannitol. Among the other reported cases of pediatric ODS, one patient received HS [5], 3 received mannitol [7, 13], and one patient did not receive any hyperosmolar therapy [12]. In the case reported by Sivaswamy et al. [12], there were no wide fluctuations in the sodium level but they noted fluctuations in the osmolarity. While the data are limited in the context of cerebral edema and ODS in the pediatric DKA population, osmotic changes may be responsible for these complications. The available guidelines for the management of cerebral edema in DKA [2, 3] have recommendations on the initiation of the therapy only. The recommendations are lacking on further continuation of the therapy and in the context of rebound ICP and development of SAH and ODS. This would be helpful in cases like ours, where patient was symptomatic for over 5 days with MRI showing cerebral edema on day 3. Hence we extrapolated our management based on the guidelines for TBI population [18, 19]. The rate of rise, the rate of reduction, and the upper limit of sodium and osmolarity were maintained in our case within the guidelines for CE management in cases of TBI. It is plausible that hyperosmolar therapy which increased the level of sodium and the osmolarity significantly contributed to the development of ODS in our patient. However, given the severity of cerebral edema in our patient, it is very likely that it was also a lifesaving intervention. Further larger studies and specific recommendations in terms of serum sodium, osmolarity, and maintenance of hyperosmolar therapy for the CE management in the DKA population will be helpful.

The age group in the other five reported cases of ODS ranged from 18 months to 17 years. The proposed mechanism for the development of ODS varied among the cases but overall included severe acidosis (pH < 7.0), aggressive fluid resuscitation, hyperosmolar therapy, bacteremia with possible meningitis, and wide fluctuations in glucose, sodium, or osmolarity. One patient died and the remaining four survived with full or near full recovery [5, 7, 8, 13, 14].

A larger review of pediatric ODS showed association with a wide array of underlying illnesses including head injury, liver trauma, malignancy, intracranial surgery with or without diabetes insipidus, ornithine transcarbamylase deficiency, acute gastroenteritis, and adrenal insufficiency [5].

The prognosis of CE and ODS varies from full recovery to death. ODS was historically thought to be fatal or to result in severe neurologic deficits including locked-in syndrome. However, in the last 30 years there have been increasing numbers of pediatric cases with complete or near-complete recovery [5, 7, 8, 13, 14, 30]. This shift in outcome is possibly due to improved identification and advancement in medical care.

The etiology of neurologic sequelae in our patient is most likely multifactorial. Despite these severe and rare complications, our patient made near full recovery, which is inspirational.

## Data Availability

The data used to support the findings of this case report are included within the article.
